# Complexity of Self-Gravitating Systems

**DOI:** 10.3390/e23070802

**Published:** 2021-06-24

**Authors:** Luis Herrera

**Affiliations:** Instituto Universitario de Física Fundamental y Matemáticas, Universidad de Salamanca, 37008 Salamanca, Spain; lherrera@usal.es

In recent decades many efforts have been made towards a rigorous definition of complexity in different branches of science (see [1,2,3,4,5,6,7,8,9,10,11,12] and references therein). However, despite all the work done so far, there is not yet a consensus on a precise definition.

The reason behind such interest stems from the fact that at least at an intuitive level, complexity, no matter how we define it, is a physical concept deeply intertwined with fundamental aspects of the system. In other words, we expect that a suitable definition of complexity of the system could allow us to infer relevant conclusions about its behavior.

Therefore, it is of utmost relevance to provide a precise definition of an observable quantity which allows measurement of such an important property of the system. Thus, when dealing with a situation that intuitively is judged as “complex”, we need to be able to quantify this complexity by defining an observable measuring it.

Among the many definitions that have been proposed so far, most of them resort to concepts such as information and entropy, and are based on the intuitive idea that complexity should, somehow, measure a basic property related to the structural characteristics of the system.

This Special Issue of Entropy is devoted to the discussion of the possible definition of the complexity of self-gravitating systems and their applications.

An extension of the definition of complexity based on the work developed by López-Ruiz and collaborators [7,10] has already been proposed for self-gravitating systems in [13,14,15,16,17,18].

However, such a definition suffers from two drawbacks, which motivated the introduction of a quite different definition which was proposed in [19] for the static spherically symmetric case, and extended further in [20] to the general full dynamic case.

The definition given in [19], although intuitively associated with the very concept of “structure” within the fluid distribution, is not related (at least directly) to information or disequilibrium; rather it stems from the basic assumption that the simplest system (or at least one of them) is represented by the homogeneous fluid with isotropic pressure. Having assumed this fact as a natural definition of a vanishing complexity system, the very definition of complexity emerges in the development of the fundamental theory of self-gravitating compact objects, in the context of general relativity.

The variable responsible for measuring complexity, which we call the complexity factor, appears in the orthogonal splitting of the Riemann tensor, and the justification for such a proposition, roughly speaking, is as follows.

For a static fluid distribution, the simplest system is represented by a homogeneous (in the energy density), locally isotropic fluid (principal stresses equal). So, we assign zero value of the complexity factor for such a distribution. Next, let us recall the concept of Tolman mass [21], which may be interpreted as the “active” gravitational mass, and may be expressed, for an arbitrary distribution, through its value for the zero-complexity case plus two terms depending on the energy density inhomogeneity and pressure anisotropy, respectively. These latter terms in turn may be expressed through a single scalar function that we call the complexity factor. It obviously vanishes when the fluid is homogeneous in the energy density, and isotropic in pressure, but also may vanish when the two terms containing density inhomogeneity and anisotropic pressure cancel each other out. Thus, as in [7], vanishing complexity may correspond to very different systems.

When dealing with time-dependent systems, we face two different problems; on the one hand, we have to generalize the concept of complexity of the structure of the fluid distribution to time-dependent dissipative fluids, and on the other hand we also have to evaluate the complexity of the patterns of evolution and propose what we consider to be the simplest of them.

In [20] it was shown that the complexity factor for the structure of the fluid distribution is the same scalar function as for the static case, which now includes the dissipative variables. As for the simplest pattern of evolution, it was shown that the homologous condition characterizes the simplest possible mode. However, as was shown later on, it may be useful to relax this last condition to enlarge the set of possible solutions, by adopting the so-called quasi-homologous condition, which was done in [22].

The axially symmetric static case has been considered in [23], while some particular cases of cylindrically symmetric fluid distributions have been studied in [24,25]. Further applications of the concept of complexity as defined in [19] may be found in [26,27,28,29,30]. Always within the context of general relativity, exact solutions for static fluid distributions endowed with hyperbolical symmetry and satisfying the condition of minimal complexity were presented in [30], while dynamic solutions endowed with hyperbolical symmetry and satisfying the condition of minimal complexity were obtained in [31], such solutions evolve in the so-called quasi-homologous regime.

The concept of complexity as defined in [19] has also been extended to other theories of gravity in [32,33,34,35,36,37,38,39,40,41,42,43,44,45,46,47,48,49,50,51].

All the above-mentioned cases concern fluid distributions (which eventually may be charged); however, the vacuum case has barely been treated. The only known example is the extension of the complexity factor as defined in [19] to the vacuum solutions of the Einstein equations represented by the Bondi metric [52]. A complexity hierarchy was established in this case, ranging from the Minkowski spacetime (the simplest one) to gravitationally radiating systems (the most complex).

## Open Issues

As follows from the comments above, we have already available a good candidate for measuring the complexity of a self-gravitating system. However, it is by no means unique and it is therefore pertinent to ask what alternative definitions to the complexity factor as defined in [19] may be proposed. In the same line of arguments, the simplest patterns of evolution assumed so far are the homologous and the quasi-homologous regimes. However, once again, it is not clear whether or not other patterns of evolution could also fit the role of the simplest pattern of evolution.

Additionally, we believe that a definition of complexity for vacuum space–time is worth considering. Finally, new exact solutions to the field equations in the context of the Einstein theory or any alternative one, would serve as a test-bed for the definition of complexity.

Based on these remarks, we propose below a list of questions which we would like to see treated in the manuscripts submitted to this Special Issue. It is of course a partial list, and it goes without saying that any manuscript devoted to a subject related to the concept of complexity of self-gravitating systems, but not mentioned in the list below, would also be welcome.

- Are there alternative definitions of complexity different from the one proposed in [19]?
- How can we extend the definition of complexity for vacuum space–time?
- Besides the homologous and the quasi-homologous regime, could we define another pattern of evolution that could qualify as the simplest one?
- Can we relate the complexity factor(s) in the non-spherically symmetric case to the active gravitational mass, as in the spherically symmetric case?
- Can we single out a specific family of exact axially symmetric static solutions satisfying the vanishing complexity factor(s) condition?
- Can any of the above solutions be matched smoothly to any vacuum Weyl solution?
- The definition of complexity proposed in [19] is not directly related to entropy or disequilibrium, although it is possible that such a link might exist after all. If so, how could such relationship be brought out?
- Could it be possible to provide a definition of the arrow of time in terms of the complexity factor?
- How is the complexity factor related to physical relevant properties of the source, in terms of stability or maximal degree of compactness?
- How does the complexity factor evolve? Do physically meaningful systems prefer vanishing complexity factors?
- Should a physically sound cosmological model have a vanishing complexity factor? Should it evolve in the homologous or quasi-homologous regime?
- The complexity factor for a charged fluid is known, but what is the complexity factor for a different type of field (e.g., scalar field?).
- How should we define the complexity factor in the context of other alternative theories of gravity that have not been considered so far?
- How can we find new solutions satisfying the vanishing complexity factor? Could we use the general methods described in [53,54,55,56,57] to obtain such solutions?
- What relevant physical features share solutions satisfying the vanishing complexity factor?
- Is there a link between the concept of complexity and some kind of symmetry (e.g., motions, conformal motions, affine collineations, curvature collineations, matter collineations, etc.)?

## References

[1] Kolmogorov A.N. (1965). Three approaches to the definition of the concept “quantity of information”. Prob. Inform. Theory J..

[2] Grassberger P. (1986). Toward a quantitative theory of self-generated complexity. Int. J. Theor. Phys..

[3] Lloyd S., Pagels H. (1988). Complexity as thermodynamic depth. Ann. Phys..

[4] Crutchfield J.P., Young K. (1989). Inferring statistical complexity. Phys. Rev. Lett..

[5] Anderson P.W. (1991). A Dialogue on the Theory of High Tc. Phys. Today.

[6] Parisi G. (1993). Statistical Physics and biology. Phys. World.

[7] López-Ruiz R., Mancini H.L., Calbet X. (1995). A statistical measure of complexity. Phys. Lett. A.

[8] Feldman D.P., Crutchfield J.P. (1998). Measures of statistical complexity: Why?. Phys. Lett. A.

[9] Calbet X., López-Ruiz R. (2001). Tendency towards maximum complexity in a nonequilibrium isolated system. Phys. Rev. E.

[10] Catalán R.G., Garay J., López-Ruiz R. (2002). Features of the extension of a statistical measure of complexity to continuous systems. Phys. Rev. E.

[11] Sañudo J., López-Ruiz R. (2008). Statistical complexity and Fisher–Shannon information in the H-atom. Phys. Lett. A.

[12] Panos C.P., Nikolaidis N.S., Chatzisavvasand K.C., Tsouros C.C. (2009). A simple method for the evaluation of the information content and complexity in atoms. A proposal for scalability. Phys. Lett. A.

[13] Sañudo J., Pacheco A.F. (2009). Complexity and white-dwarf structure. Phys. Lett. A.

[14] Chatzisavvas K.C., Psonis V.P., Panos C.P., Moustakidis C.C. (2009). Complexity and neutron star structure. Phys. Lett. A.

[15] de Avellar M.G.B., Horvath J.E. (2012). Entropy, complexity and disequilibrium in compact stars. Phys. Lett. A.

[16] de Souza R.A., de Avellar M.G.B., Horvath J.E. (2013). Statistical measure of complexity in compact stars with global charge neutrality. arxiv.

[17] de Avellar M.G.B., Horvath J.E. (2013). Entropy, Disequilibrium and Complexity inCompact Stars:An information theory approach to understandtheir Composition. arxiv.

[18] de Avellar M.G.B., de Souza R.A., Horvath J.E. (2014). Information theoretical methods as discerning quantifiers of the equations of state of neutron stars. Phys. Lett. A.

[19] Herrera L. (2018). New definition of complexity for self-gravitating fluid distributions: The spherically symmetric, static case. Phys. Rev. D.

[20] Herrera L., Di Prisco A., Ospino J. (2018). Definition of complexity for dynamical spherically symmetric dissipative self-gravitating fluid distributions. Phys. Rev. D.

[21] Tolman R. (1930). On the Use of the Energy-Momentum Principle in General Relativity. Phys. Rev..

[22] Herrera L., Prisco A.D., Ospino J. (2020). Quasi-homologous evolution of self-gravitating systemswith vanishing complexity factor. Eur. Phys. J. C.

[23] Herrera L., Prisco A.D., Ospino J. (2019). Complexity factors for axially symmetric static sources. Phys. Rev. D.

[24] Sharif M., Butt I. (2018). Complexity Factor for Charged Spherical System. Eur. Phys. J. C.

[25] Sharif M., Butt I. (2018). Complexity factor for static cylindrical system. Eur. Phys. J. C.

[26] Sharif M., Butt I. (2019). Electromagnetic effects on complexity factor for static cylindrical system. Chin. J. Phys..

[27] Casadio R., Contreras E., Ovalle J., Sotomayor A., Stuchlik Z. (2019). Isotropization and change of complexity. Eur. Phys. J. C.

[28] Khan S., Mardan S., Rehman M. (2019). Framework for generalized polytropes with complexity. Eur. Phys. J. C.

[29] Sharif M., Tariq S. (2020). Complexity factor for charged dissipative dynamical system. Mod. Phys. Lett. A.

[30] Herrera L., Prisco A.D., Ospino J. (2021). Hyperbolically symmetric static fluids: A general study. Phys. Rev. D.

[31] Herrera L., Prisco A.D., Ospino J. (2021). Symmetry.

[32] Abbas G., Nazar H. (2018). Complexity factor for static anisotropic self-gravitating. Eur. Phys. J. C.

[33] Abbas G., Nazar H. (2018). Complexity factor for anisotropic source in non-minimal coupling metric f (R) gravity. Eur. Phys. J. C.

[34] Sharif M., Majid A. (2019). Complexity factor for static sphere in self-interacting Brans??Dicke gravity. Chin. J. Phys..

[35] Nazar H., Abbas G. (2019). Complexity factor for dynamical spherically symmetric fluid distributions in gravity. Int. J. Geom. Methods Mod. Phys..

[36] Sharif M., Majid A. (2019). Complexity factors for static axial system in self-interacting Brans–Dicke gravity. Int. J. Geom. Methods Mod. Phys..

[37] Abbas G., Ahmed R. (2019). Complexity factor for a class of compact stars in f(R,T) gravity. Astron. Space Sci..

[38] Sharif M., Majid A., Nasir M. (2019). Complexity factor for self-gravitating system in modified Gauss–Bonnet gravity. Int. J. Mod. Phys. A.

[39] Zubair M., Azmat H. (2020). Complexity analysis of dynamical spherically-symmetric dissipative self-gravitating objects in modified gravity. Int. J. Mod. Phys. D.

[40] Yousaf Z., Bhatti M., Naseer T. (2020). Study of static charged spherical structure in f(R, T, Q) gravity. Eur. Phys. J. Plus.

[41] Zubair M., Azmat H. (2020). Complexity analysis of Cylindrically Symmetric Self-gravitating Dynamical System in f(R,T) Theory of Gravity. Phys. Dark. Univ..

[42] Yousaf Z., Bhatti M., Naseer T. (2020). New Definition of Complexity Factor in f(R, T, R_μν_T^μν^) Gravity. Phys. Dark. Univ..

[43] Abbas G., Nazar H. (2020). Complexity factors for static anisotropic axially symmetric fluid distributions in f(R) gravity. Int. J. Geom. Methods Mod. Phys..

[44] Yousaf Z., Bhatti M., Hassan K. (2020). Complexity for self-gravitating fluid distributions in f(G, T) gravity. Eur. Phys. J. Plus.

[45] Yousaf Z., Bhatti M., Naseer T., Ahmad I. (2020). The measure of complexity in charged celestial bodies in f(R, T, R_μν_T^μν^) gravity. Phys. Dark. Univ..

[46] Yousaf Z. (2020). Definition of complexity factor for self-gravitating systems in Palatini f(R) gravity. Phys. Scr..

[47] Yousaf Z., Khlopov M.Y., Bhatti M.Z., Naseer T. (2020). Influence of modification of gravity on the complexity factor of static spherical structures. Month. Not. R. Astron. Soc..

[48] Yousaf Z., Bhatti M., Naseer T. (2020). Evolution of the charged dynamical radiating spherical structures. Ann. Phys..

[49] Yousaf Z., Bhatti M., Naseer T. (2020). Measure of complexity for dynamical self-gravitating structures. Int. J. Mod. Phys. D.

[50] Sharif M., Majid A. (2020). Evidence for tt¯tt¯ production in the multilepton final state in proton-proton collisions at √s=13 TeV with the ATLAS detector. Eur. Phys. J. C.

[51] Yousaf Z., Bamba K., Bhatti M.Z., Hassan K. (2021). Measure of complexity in self-gravitating systems using structure scalars. New Astron..

[52] Herrera L., Prisco A.D., Carot J. (2019). Complexity of the Bondi Metric. Phys. Rev. D.

[53] Thirukkanesh S., Maharaj S.D. (2009). Radiating relativistic matter in geodesic motion. J. Math. Phys..

[54] Thirukkanesh S., Maharaj S.D. (2010). Mixed potentials in radiative stellar collapse. J. Math. Phys..

[55] Ivanov B. (2011). Self-gravitating spheres of anisotropic fluid in geodesic flow. Int. J. Mod. Phys. D.

[56] Ivanov B. (2016). All solutions for geodesic anisotropic spherical collapse with shear and heat radiation. Astrophys. Space Sci..

[57] Ivanov B. (2016). A different approach to anisotropic spherical collapse with shear and heat radiation. Int. J. Mod. Phys. D.

